# Immunoinformatics Comes of Age

**DOI:** 10.1371/journal.pcbi.0020071

**Published:** 2006-06-30

**Authors:** Bette Korber, Montiago LaBute, Karina Yusim

**Affiliations:** National Center for Biotechnology Information, United States of America

## Abstract

With the burgeoning immunological data in the scientific literature, scientists must increasingly rely on Internet resources to inform and enhance their work. Here we provide a brief overview of the adaptive immune response and summaries of immunoinformatics resources, emphasizing those with Web interfaces. These resources include searchable databases of epitopes and immune-related molecules, and analysis tools for T cell and B cell epitope prediction, vaccine design, and protein structure comparisons. There is an agreeable synergy between the growing collections in immune-related databases and the growing sophistication of analysis software; the databases provide the foundation for developing predictive computational tools, which in turn enable more rapid identification of immune responses to populate the databases. Collectively, these resources contribute to improved understanding of immune responses and escape, and evolution of pathogens under immune pressure. The public health implications are vast, including designing vaccines, understanding autoimmune diseases, and defining the correlates of immune protection.

## Introduction 

### 

#### The adaptive immune response.

The immune system is the body's defense against infectious organisms and other foreign agents. The first line of defense is innate immunity, rapid nonspecific responses that allow recognition of conserved signature structures present in many microorganisms, such as lipopolysaccharides in bacterial cell walls or proteins in flagella [1]. The second line of defense is the adaptive immune response, tailored to an individual threat. An infected host mounts an immune response specific to an infectious agent; after the infection is resolved, memory cells persist that enable a more rapid and potent response if the infectious agent is encountered again.

The adaptive immune response has two major arms: the cellular immune response of T lymphocytes, and the humoral immune response of antibody-secreting B lymphocytes. In both cases the immune response is stimulated by receptor recognition of a specific small part of an antigen known as an epitope. Antibodies generally recognize intact proteins. B cell epitopes can be linear, contiguous amino acids, or they can be discontinuous amino acids that are brought together spatially in folded proteins. Discontinuous epitopes are defined through mutagenesis, competition experiments, modeling, or through cocrystallization or modeling of protein structure and docking [2]. Even linear B cell epitopes are often conformation-dependent, and antibody-antigen interactions are improved when the epitope is displayed in the context of the folded protein.

In contrast, T cell epitopes are short linear peptides that are cleaved from antigenic proteins, although T cell epitope generation by protein splicing is also observed [3]. T cell epitopes are presented in the context of major histocompatibility complex (MHC) proteins, or, in case of humans, human leukocyte antigen (HLA) class I or class II molecules. Epitope presentation depends on both MHC-peptide binding and T cell receptor (TCR) interactions [4,5]. MHC proteins are highly polymorphic, and each binds to a limited set of peptides. Thus the particular combination of MHC alleles present in a host limits the range of potential epitopes recognized during an infection. The conformation of a T cell epitope embedded in an MHC protein is critical for TCR recognition [6,7].

Two fundamental types of T cells are distinguished by expression of CD8 and CD4 proteins, which dictate whether a T cell will recognize epitopes presented by class I or class II molecules, respectively. Underlying this high-level bifurcation is a complex array of other functional markers. A key effector function of CD8^+^ T cells is cytolytic activity resulting in apoptosis of virally infected cells [8], which depends upon the CD8^+^ T cell's previous exposure to antigen and activation state [9]. The primary function of CD4^+^ T cells is to produce cytokines that regulate the rest of the immune response. These functions are not exclusive, however—CD4^+^ T cells can induce cytolysis [10], and CD8^+^ T cells can secrete immunoregulatory factors.

CD4^+^ T cell epitopes are processed after encapsulation by antigen-presenting cells in membrane-bound vesicles, where they are degraded by proteases into the peptide fragments that bind to MHC class II proteins. Then they are delivered to the cell surface, where class II-peptide complexes can be recognized by the CD4^+^ TCRs [5]. In contrast, CD8^+^ T cells generally recognize viral or self antigens expressed from within a cell [11], proteins that are cleaved into short peptides in the cytosol by the immunoproteasome [12] at the C-terminal end of the peptide [13]. The N terminus is later trimmed by proteases in endoplasmic reticulum [14]. After cleavage, peptides are translocated by the transporter associated with antigen processing (TAP) into the endoplasmic reticulum for loading onto HLA class I molecules [12,15], although other transport pathways can be used [16]. The MHC class I-peptide complex is then presented on the cell surface, allowing recognition by epitope-specific TCRs on CD8^+^ T cells [5,12].

Both B cell and T cell epitopes are constrained by sequence specificity, and mutations within and external to epitopes can result in immune escape. Obviously, mutations within an epitope can directly impact antibody-antigen interactions or epitope-MHC and TCR interactions. Mutations outside of the epitope can inhibit antibody binding through conformational changes, or inhibit proper cleavage and processing of T cell epitopes [17,18]. TAP also binds peptides somewhat selectively [19]. While there is a predilection for certain peptides to be processed for MHC binding and presentation, processing steps must be general enough to accommodate a wide variety of potential epitopes so as to not excessively constrain T cell immunity.

Pathogen- and cancer-related immune responses are being characterized at a remarkable pace, with precise mapping of well-characterized epitopes and increasing use of full genetic typing of HLA-epitope presenting molecules, characterization of accompanying crystal structures, and definitions of escape mutations. As these elements are defined piece-by-piece in the literature, it becomes increasingly valuable to assemble the data into searchable databases and to provide computational tools to assist in interpretation of this complex information. Defining epitope sequence specificity (including cleavage and transport signals and MHC binding) presents a tantalizing problem for computational biologists. The predictive amino acid patterns associated with these events are subtle, requiring sophisticated pattern recognition methods to infer directly from protein sequences which peptides have the potential to become epitopes. The complexity is compounded by the fact that recognition patterns might not be encoded by the contiguous primary sequence, but rather in local three-dimensional structure. The response to this challenging problem has resulted in an abundance of Web-based methods enabling the exploration of immunologically relevant data from a variety of perspectives. This review summarizes a sampling of particularly useful and user-friendly Web-based computational tools and searchable databases. The computational methods and databases are described and referenced in the text, and Web links are provided in summary tables. As a cautionary note, the authors have not directly tested that the functions contained in these resources will produce meaningful results, nor have we done systematic comparisons of the output of the different analysis tools; users would benefit by reading the primary literature regarding the different analyses methods if they decide to use one or more in their own work.

#### Tools for predicting potential T cell epitopes in protein sequences.

The most thoroughly studied step of T cell epitope generation is peptide binding to MHC molecules, and the Web-based databases that include peptide-MHC data enable binding predictions. The MHCPEP database [20], for example, contains 13,000 MHC-binding peptides. Each entry contains the peptide sequence, its MHC specificity and, when available, experimental methods, observed activity, binding affinity, source protein, anchor positions, and references. This database, however, has been static since 1998. MHCBN [21] includes 18,790 MHC-binding peptides, 3,227 MHC-nonbinding peptides, 1,053 TAP binders and nonbinders, and 6,548 T cell epitopes. A beta-version of the new Immune Epitope Database and Analysis Resource (IEDB) has recently come online that will focus on epitopes in potential bioterrorism agents or emerging infectious diseases [22]. More databases are available, and some are discussed below together with relevant prediction tools.

**Figure 1:**
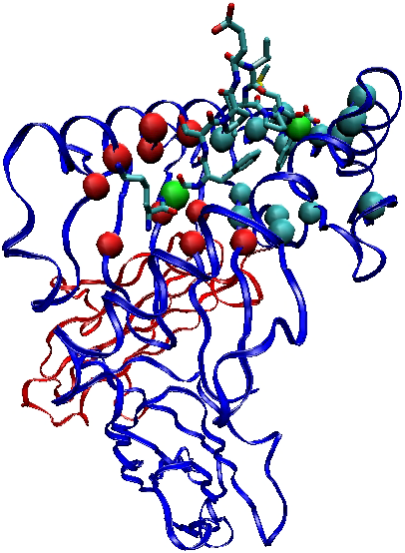
Interaction of an Epitope with an MHC Class I Protein Ribbon representation of the 1.65 Å resolution X-ray crystal structure of the MHC I allele B*5703 in complex with the KAF-11 peptide (KAFSPEVIPMF) derived from the HIV-1 p24 capsid protein. The blue ribbon indicates the alpha chain, the red chain is beta-2 microglobulin, and the molecule in the binding cleft is the antigenic peptide. The red and blue-green spheres mark the alpha carbons of the canonical peptide-binding B- and F-pocket residues, respectively. The green spheres represent the alpha carbons of the peptide anchor residues at P2 and P11.

Peptide-MHC binding is the most predictable aspect of T cell epitope generation. MHC class I and class II genes are highly polymorphic, and the majority of their variable positions are located in binding pockets that restrict peptide interactions to those with particular amino acids at characteristic positions (Figure 1); the set of amino acids that are well tolerated in these binding pockets are called anchor motifs. The search for epitopes in full-length proteins or within the context of a reactive peptide can be narrowed through a search for MHC-appropriate anchor motifs. Primary HLA class I anchor positions are generally located at the C terminus and a middle position of a peptide; as optimal epitope lengths vary between 8 and 12 amino acids long, the spacing between these two positions varies [23,24]. The first MHC allele-specific motifs were defined for murine class II molecules [25]. Tracking anchor motifs patterns alone was soon found to be of limited predictive value [26], while including more extensive binding patterns using quantitative matrices representing the frequency and weight of every amino acid in every position enabled the prediction of epitope locations in protein sequences with somewhat greater [24,27–31], although still limited [32], accuracy.

For many MHC alleles, both simple and extended motifs are characterized and used to predict potential epitopes. For example, the SYFPEITHI database [33] contains extensive information on MHC class I and class II anchor motifs and binding specificity, and includes more than 4,500 entries of MHC proteins and aligned sequences of their epitopes and natural ligands, with source proteins, organisms, and publication references for each peptide. The SYFPEITHI epitope prediction server [33] uses a frequency-based scoring system for every amino acid position within a peptide. The SYFPEITHI database allows, through examination of aligned peptides known to bind the HLA molecules, appreciation of the relative level of conservation of anchor motifs, as well as the number of peptides that bind despite imperfect motifs.

The Los Alamos HIV/HCV databases offer a simple tool (MotifScan) for identifying HLA anchor-binding motifs in query proteins, highlighting them on a protein or protein alignment [34,35]. This tool is based on motif libraries included at the SYFPEITHI site, assembled by S. Marsh and colleagues [23,24], and motifs extracted from the primary literature. The more sophisticated MHC-peptide binding prediction approaches have generally been applied to limited numbers of MHC proteins, so MotifScan provides a more comprehensive, but less reliable, exploration of potential HLA-binding peptides. The input protein sequences can be automatically uploaded from predefined sets of HIV or HCV proteins, or the user can input any protein sequence or sequence alignment. MotifScan is taken one step further for HIV and HCV through the Epitope Location Finder (ELF) [36], where HLA anchor motifs are mapped onto proteins or peptides in conjunction with known epitopes taken from extensive database listings of class I HIV and HCV T cell epitopes and their presenting HLAs [37,38]. Currently the HIV CD8^+^ T cell epitope database contains 3,150 entries describing 1,600 distinct MHC class I-epitope combinations (a single epitope can have multiple entries); the HCV database contains 510 entries describing 250 distinct MHC class I–epitope combinations. These databases include detailed biological information regarding the response to the epitope, including its impact on long term survival, common escape mutations, and whether an epitope is recognized in early infection; links to the primary literature; and curated alignments summarizing the epitope's global variability.

A central assumption of the traditional prediction methods based on motif frequencies is that each position contributes independently to binding. Interactions at one site, however, can affect interactions in another site [27,39]. Statistical classifiers such as Hidden Markov Models have better success rates at MHC-binding predictions, and machine learning methods such as artificial neural networks and support vector machines can recognize nonlinear sequence-dependent correlated effects in MHC binding. Machine learning methods as well as statistical methods are also useful for defining characteristic sequences related to TAP binding, and for addressing the complexity of proteasome cleavage [40–47]. These methods, however, require large numbers of well-characterized peptides as training sets [32]. One comparative analysis suggested that motifs gave the most accurate MHC-binding predictions with limited data, but as the data increases, machine learning methods become more reliable predictors [48]. In another comparative study, a support vector machine outperformed other methods [40]. Both motif-based and machine learning methods for prediction of different steps of T cell epitope generation are available (Table 1) [49], often offered in combination with databases of MHC-ligand interactions (Table 2). Below we discuss some of the Web sites that are particularly helpful for T cell epitope prediction, many of which incorporate all three elements: immunoproteasome cleavage, TAP binding, and MHC binding.

**Table 1:**
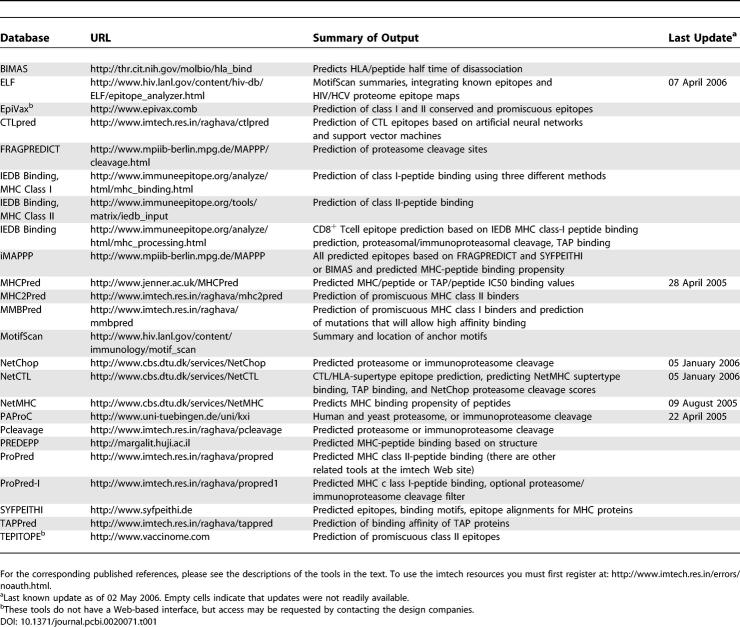
Web-Based Interactive Tools for T Cell Epitope Prediction

**Table 2:**
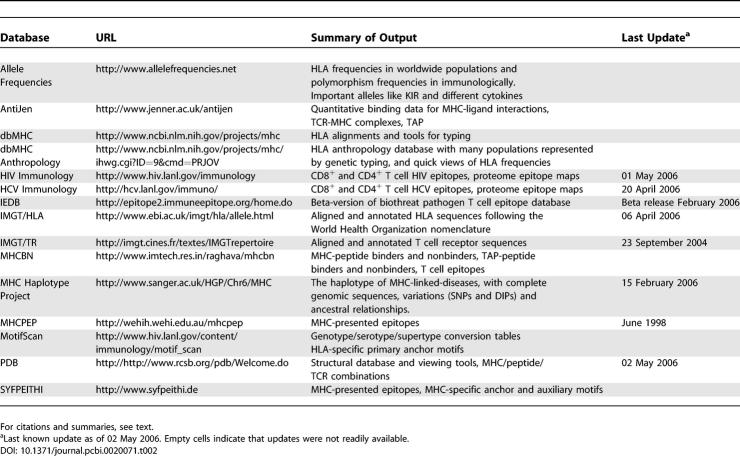
T Cell-Related Immunological Databases and Tables

The Edward Jenner Institute for Vaccine Research maintains the AntiJen database, which contains quantitative experimental binding data for peptides that bind to MHC, TAP, TCR-MHC complexes, T cell epitopes, and B cell epitopes; it also offers data on immunological protein-protein interactions. It includes more than 24,000 entries. The MHCPred [50,51] tool predicts the energetics of protein-ligand interactions related to the free energy of binding, and takes into account individual amino acids and contributions from side chain-side chain interactions, allowing peptide-MHC and peptide-TAP binding predictions. This site also allows the prediction of high affinity peptides by comparing the predicted binding affinities of the original and the mutated peptides. PREDEPP [52,53] relies on the structural conservation and interactions observed in crystal structures of peptide-MHC complexes. A peptide's compatibility for binding is evaluated statistically by pairwise potentials. The Web site also predicts proteasomal cleavage sites [54].

The BIMAS tool [31,55] ranks potential peptides based on a predicted half-time of disassociation from HLA class I molecules, based on coefficient tables deduced from the published literature. The Max Planck Institute for Infection Biology offers MAPPP software [56] that combines either BIMAS or SYFPEITHI MHC-binding prediction with the proteasome cleavage software FRAGPREDICT [57]. FRAGPREDICT predicts potential proteasomal cleavage sites based on a combination of two algorithms. A statistical analysis of cleavage-determining amino acid patterns is performed [57], followed by predictions of major proteolytic fragments based on a kinetic model of the 20S proteasome describing the time-dependent digestion of smaller (up to 40 residues long) peptide substrates [58].

The following three suites of tools allow MHC/class I epitope prediction through a combination of cleavage prediction, TAP binding, and MHC binding. The Center for Biological Sequence Analysis offers the NetChop tool [44,59] for predicting proteasomal or immunoproteasomal cleavage using a nonlinear neural network, trained on in vitro experimental cleavage data or MHC class I ligand data, respectively. NetMHC [60–62] predicts binding of peptides to HLA supertypes (groups of HLA proteins that are likely to cross-present epitopes because of similarity in allowed binding motifs) or to 120 individual HLA alleles, using artificial neural networks. NetCTL [63,64] predicts epitopes by combining predictions of peptide-HLA-supertype binding (NetMHC), proteasomal C-terminal cleavage (NetChop), and TAP transport efficiency using a weight-matrix based method [65]. The Bioinformatics Centre Institute of Microbial Technology has also developed a suite of servers [21,40–42,66,67] designed for predicting immunologically interesting features in antigen sequences. ProPred1 and ProPred, along with a series of related programs using different strategies, predicts specific MHC-binding peptides in proteins [67,68]. Promiscuous binders can be predicted using a support vector machine by MHC2Pred for MHC class II, or quantitative matrices by MMBPred for MHC class I [69]. Pcleavage uses a support vector machine to predict proteasomal cleavage based on in vitro data, or immunoproteasomal cleavage data based on MHC class I ligand data [42]. TAPPred predicts binding to TAP [41]. CTLpred predicts CTL epitopes in an antigen sequence by combining the processing and binding prediction methods [40]. IEDP also offers a suite of tools for T cell epitope prediction. Their peptide-MHC class I binding prediction tool allows the options of using an artificial neural net, average relative binding [70], or a stabilized matrix method [71]. A comparison of the accuracy of these methods is underway by the IEDP team. These three methods also use the average binding method for the prediction of MHC class II peptide binding [70]. Their MHC class I-peptide binding prediction can be combined with immunoproteasome cleavage [72] and TAP transport predictions [65], to predict MHC class I epitopes.

Many of the sites listed are convenient for large-scale calculations. Some, for example SYFPEITHI and MHCPred, allow one to incorporate multiple HLA alleles for epitope prediction, while others, such as NetChop, NetMHC, NetCTL, FRAGPREDICT, and IEDP tools allow one to upload protein alignments. MotifScan, MAPPP, and the ProPred series allow both. These methods are currently being applied to peptide vaccine design and can be used to identify epitopes that have the desirable properties of promiscuous presentation by many HLAs and relative conservation [69,73,74]. We have recently taken a very different approach to T cell vaccine design and developed a computational method for designing polyvalent protein cocktails that provide maximum peptide coverage (where peptides are set to a user-specified length, for example nine amino acids) in a population of diverse proteins [75]. The mosaic proteins we create resemble real proteins, as they are assembled using a genetic algorithm by in silico homologous recombination of natural strains, and sets of mosaics are created based on the optimizing their combined population coverage. While no Web interface has yet been built for this code, the two related programs are freely available. One program enables an exploration of the peptide coverage in any set of natural proteins by a prototype vaccine strain or combinations of strains, while the other designs sets of mosaic proteins for a polyvalent vaccine that will maximize population coverage. These tools could be applied to any variable pathogen for vaccine design, or used to design sets of reagents to probe the immune response.

#### HLA-related databases and Web services.

The number of genetically defined MHC and HLA alleles continues to expand, with a corresponding evolving and expanding nomenclature. The European Bioinformatics Institute maintains the IMGT/HLA sequence database [76], which includes HLA allele listings as defined in the World Health Organization Nomenclature Committee Reports. The reports include previous designations, accession numbers, references, and information on the source of the allele. This Web site has sequences and alignments from HLA class I and II loci, from the related MICA and MICB loci and from TAP1 and TAP2. To find Protein Data Bank (PDB) structures of MHC alleles in complex with peptides and/or the TCR domain, one easy method is to perform a BLAST search using the MHC alpha chain on PDB itself. There are about 100 available structures of MHCs in complex with peptides (mostly A alleles for MHC class I), and 20 of MHC, peptide, and TCR complexes (mostly involving HLA A2-related alleles).

The National Center for Biotechnology Information (NCBI) maintains dbMHC [77], which includes summaries of the genetic organization of the HLA region, genetic sequence alignments, and tools for HLA typing. It also houses the HLA anthropology database, where individual allele and haplotype frequencies can be retrieved from many different populations, nations, or geographic areas. The Allele Frequencies in Worldwide Populations project also offers summaries of HLA frequencies, as well as polymorphisms in cytokines and KIR alleles. The Sanger MHC haplotype project offers information on MHC related disease haplotypes, sequences, polymorphisms, and ancestral relationships [78,79].

#### Tools to assist the experimental T cell immunologist.

Experimental T cell response mapping efforts recently have been scaling up, including additions of variant peptides to better probe responses to variable pathogens and extensions of T cell response mapping studies to span the full proteome of pathogens for large study populations (for one example of a population study incorporating Elisot mapping of T cell responses to HIV, see [80]). Complete datasets for several of these large T cell peptide response studies for HIV are available (http://www.hiv.lanl.gov/content/immunology/hlatem/index.html). These efforts have led the HIV/HCV database team to develop computational tools to facilitate study design and analyses of experimental data of this nature. These tools could, for the most part, be applied to any pathogen or protein. PeptGen [81] enables a user to design overlapping peptide sets of any length and overlap, using a single sequence or an alignment if a variable pathogen is being studied and peptide variants are desired. If an alignment is used, insertions or deletions in the sequence are handled sensibly, and a ready-for-ordering peptide list is created, organized so that identical peptides between need only be ordered once. If a population with known HLA typing is screened, for example by EliSpot, Hepitope allows a rapid search for HLAs that are enriched among people that react with each peptide in the study, and provides anchor motif searches for the enriched HLAs. For HIV- or HCV-related studies, ELF [36] can be combined with Hepitope to map previously described CD8^+^ T cell epitopes onto a reactive peptide.

There is growing interest in defining and comparing HLA allele frequencies in study populations where vaccine trials are planned; thus we have made a suite of tools to compare HLA frequencies in two populations, to identify alleles in linkage disequilibrium, and to fill in estimates of missing HLA information if full genetic typing is not feasible (Note: we will add a URL if the beta-version is ready in time, and delete this section otherwise). Because of the high cost of genetic HLA typing, although it is desirable, the reality is that often only partial HLA genetic typing of key alleles is available. A partially described data set could provide the basis for informed guesses of the HLA genotypes superimposed onto two-digit typing—for example, by utilizing available four-digit data genetic typing data at dbMHC for different populations or a cohort subset that is fully genetically typed. Thus we have created a computational tool in which four-digit HLA allele designations are estimated from a combination of two-digit and four-digit HLA typing data. A maximum likelihood probability is assigned to each four-digit estimate, based on a combination of allele frequencies in the population and linkage disequilibrium patterns.

#### Tools for predicting B cell epitopes and related Internet resources.

The conformational aspects of antibody binding complicates the problem of B cell epitope prediction, making it less tractable than T cell epitope prediction. Indeed, Blythe and Flower [82] recently undertook an exhaustive assessment of amino acid propensity scales using the AntiJen B cell epitope database, and even the best combinations performed only marginally better than random [83]. If one wishes to explore antigenic propensity using traditional methods, however, IEDB provides tools for predicting five features that have been proposed to relate to B cell antigenicity, including beta turn prediction [84], surface accessibility [85], flexibility [86], and hydrophilicity [87]; it also includes an antigenicity predictor based on amino acid frequencies in antigenic domains and chemistry [88]. An alternative strategy for predicting linear B cell epitopes, ABCpred, uses a neural network trained and tested on the BCIPEP B cell epitope database [66].

Although antibody epitope prediction is difficult, many other antibody-specific resources are available on the Web (Table 3). If the variable region sequence of a monoclonal antibody is obtained, ABcheck [89] enables a rapid crosscheck against the Kabat antibody database to identify unusual residues that might be a sequencing artifact. (As a historical aside, the Kabat database was an early immunological database compiled to provide researchers with a comprehensive comparison of antibody sequences. It was available as a book long before the Internet enabled Web-searchable molecular databases, at a time when GenBank, a resource that originated at Los Alamos National Laboratory, was still in its early, groundbreaking stages. GenBank eventually moved to the National Library of Medicine. Similarly, the Los Alamos HIV database, the first pathogen-specific sequence database, was initially available only as a book of aligned viral sequences.) The sequence could then be submitted to DNAPLOT, alignment software that enables rearranged V genes to be reliably assigned to their closest V, D, and J segment germline counterparts. The most comprehensive data for crystallographic structures can be found at the molecular modeling database (MMDB) [90], summaries of antibody crystal structures are maintained at SACS [91], and both structures and alignments are available through the antibody group (ABG). The ImMunoGeneTics (IMGT) database provides annotated listings and alignments of both immunoglobulins and TCR binding regions [92,93] .

**Table 3:**
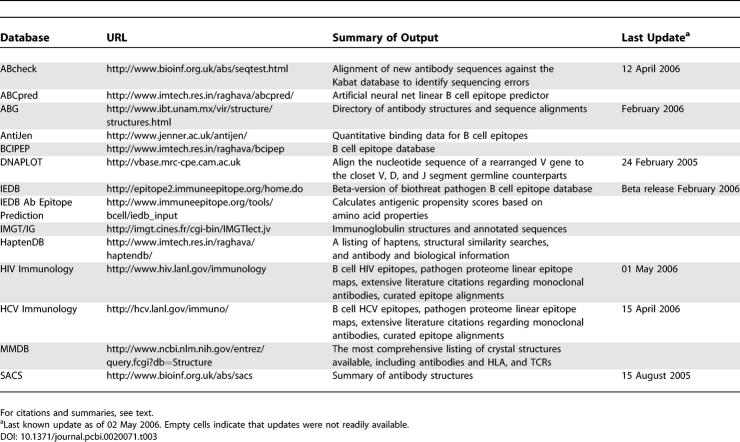
B Cell/Antibody-Related Databases and Analysis Tools

We maintain comprehensive Web-searchable databases of pathogen-specific HIV [37] and HCV antibodies [38]. These are listings of monoclonal and polyclonal responses to the proteomes of these pathogens, including information regarding epitope location and variation, escape mutations, structure, biological impact of antibody responses, keywords, and links to PubMed. The HIV database currently contains 1,273, and the HCV database 120, unique antibody entries. Antibody entries are associated with multiple publications; for some of the more intensively studied HIV neutralizing monoclonal antibodies, more than 130 papers are cited, each with a brief summary of what was learned about the specific antibody in that paper. It is difficult to track a given monoclonal antibody in the literature by other means, as often many antibodies are used in a single study so are not named in an abstract. To compound the problem, the name of a monoclonal antibody often “mutates” as it is exchanged between different labs, so is not readily searchable by traditional means.

## Discussion

This review is intended as a portal to some of the most useful online immunological software and searchable databases. This is a rapidly expanding area—experimental advances have moved immunology into population-based studies and simultaneously have brought us to the brink of comprehensively characterizing an individual's immune response to infection. Extensive listings of T cell epitopes and HLA-binding peptides, as well as peptides that do not bind, have been an invaluable resource for motif resolution and epitope prediction. Epitope prediction in turn facilitates detection of new epitopes, vaccine design, site-directed mutagenesis (to make proteins less immunogenic), potential autoantigen identification, and the design of immune-based cancer therapies. Given the compelling nature of the problem and its suitability for computational methods, many scientists have developed interesting alternative approaches to epitope prediction in silico, and have made their methods freely available through the Web (Table 1). We applaud this effort, but have the nagging concern that as the number of epitopes defined after an initial computational prediction prescreening grows, the resulting sets of experimentally defined epitopes may bias subsequent predictors in ways that traditional protein scanning with overlapping peptides would not.

Promiscuous HLA presentation and epitope prediction offers one sensible strategy for the creation of T cell vaccines [69,73,74]. Alternatively, a rational epitope-informed peptide vaccine design can utilize the data in specialized pathogen-specific databases to focus on epitopes with the most biological promise to be beneficial [94]. Finally, for a highly variable pathogen, we are trying approaches intended to improve the coverage of potential epitopes in the population, for example by using a single consensus or ancestral sequence [95–97] or a computationally designed polyvalent vaccine that will maximize epitope coverage [75].

Understanding the impact of host immune-pathogen interactions on pathogen evolution, pathogenesis, and immunogen design depends on coordinated global efforts to gather and share data and requires the combined expertise of experimental and computational scientists. Only through this type of cooperation will we fully harvest the knowledge implicit in the data. The computational tools presented here are not yet ready to supplant experiment, rather they should assist in experimental design and interpretation of data. We clearly do not know all of the rules yet, for instance in peptide-MHC binding, and key questions such as what determines immunodominance in T and B cell responses are still unanswered. Yet the range and power of the tools already available through the Internet, many representing global networks and collaboration, is a testimony to the substantial progress we have made in facing emerging infectious diseases and potential biothreats with broader and deeper collective knowledge. 

## Supporting Information

### Accession Numbers

The Protein Data Bank (http://www.rcsb.org/pdb) accession number of HIV-1 p24 capsid protein is 2BVO.

## Abbreviations

HLA: human leukocyte antigen
MHC: major histocompatibility complex
TAP: transporter associated with antigen processing
TCR: T cell receptor
